# Examining the Association Between Nursing Home Leadership Turnover and Resident Satisfaction

**DOI:** 10.1093/geroni/igaf122.3930

**Published:** 2025-12-31

**Authors:** Jenny Kwon, John Bowblis

**Affiliations:** University of California, San Francisco, San Francisco, California, United States; Miami University, Oxford, Ohio, United States

## Abstract

Leadership stability in nursing homes (NHs) plays a critical role in shaping residents’ care experience. However, the impact of nursing home administrator (NHA) and director of nursing (DON) turnover on resident satisfaction has not been thoroughly examined. This study investigates the relationship between NHA/DON turnover and resident satisfaction scores. An aggregate dataset of Ohio NHs in 2017 was constructed (N = 752) by merging five data sources: The Ohio Nursing Home Resident Satisfaction Survey, the Ohio Biennial Survey of Long-Term Care Facilities, the Certification and Survey Provider Enhanced Reports, Medicaid Cost Report, and LTCFocus. The outcome variables were eight resident satisfaction scores: overall satisfaction score and seven individual domain scores. The scores were continuous variables, with higher scores indicating greater resident satisfaction (range: 0-100). Leadership turnover was categorized into four predictors: one NHA turnover, two or more NHA turnover, one DON turnover, and two or more DON turnover over the past three years. Multiple linear regressions, including control variables, were conducted via SAS. Results show that two or more DON turnover is associated with a lower overall resident satisfaction score by 1.14% points (p < .05) and lower scores of Environment and Caregivers domains. However, NHA turnover is not statistically associated with overall resident satisfaction score, while positively associated with two domain scores. The findings of this study highlight the critical role of DON stability in improving resident satisfaction and suggest that having one leadership turnover may not compromise NH quality; however, frequent turnover, particularly among DONs, could be detrimental.

